# Modeling Chemical Reactions by QM/MM Calculations: The Case of the Tautomerization in Fireflies Bioluminescent Systems

**DOI:** 10.3389/fchem.2018.00116

**Published:** 2018-04-17

**Authors:** Romain Berraud-Pache, Cristina Garcia-Iriepa, Isabelle Navizet

**Affiliations:** Université Paris-Est, Laboratoire Modélisation et Simulation Multi Echelle, MSME, UMR 8208 CNRS, UPEM, Marne-la-Vallée, France

**Keywords:** oxyluciferin, TD-DFT, molecular dynamics, QM/MM, keto-enol tautomerization, emission spectra, bioluminescence

## Abstract

In less than half a century, the hybrid QM/MM method has become one of the most used technique to model molecules embedded in a complex environment. A well-known application of the QM/MM method is for biological systems. Nowadays, one can understand how enzymatic reactions work or compute spectroscopic properties, like the wavelength of emission. Here, we have tackled the issue of modeling chemical reactions inside proteins. We have studied a bioluminescent system, fireflies, and deciphered if a keto-enol tautomerization is possible inside the protein. The two tautomers are candidates to be the emissive molecule of the bioluminescence but no outcome has been reached. One hypothesis is to consider a possible keto-enol tautomerization to treat this issue, as it has been already observed in water. A joint approach combining extensive MD simulations as well as computation of key intermediates like TS using QM/MM calculations is presented in this publication. We also emphasize the procedure and difficulties met during this approach in order to give a guide for this kind of chemical reactions using QM/MM methods.

## Introduction

Chemical reactions are ubiquitous in biology, controlling a wide variety of fundamental biological processes such as photosynthesis, the process of vision, bioluminescence, among others (Hales, 1976; Adam, 1982; Metzler and Metzler, 2001; Palczewski, 2012). In this regard, a full understanding of the underlined chemical mechanisms is crucial. Only then, the factors that govern the specificity and efficiency of these biological processes can be determined.

In order to get insight into the mechanism and intermediates, some experimental methodologies can be used such as spectroscopic techniques, mutagenesis experiments and kinetic evaluations (Meister, 1983; Zscherp and Barth, 2001; Frey and Hegeman, 2007). However, in general many chemical questions remain unanswered from an experimental point of view and hence, the modeling of the system and the mechanism by computational chemistry is needed (Becker et al., 2001; Mulholland, 2005; van der Kamp et al., 2008).

Regarding to the modeling, quantum mechanics/molecular mechanics (QM/MM) methods are the state-of-the-art computational techniques to study chemical reactions and to compute electronic properties in complex environments as biomolecular systems (Senn and Thiel, 2007, 2009; Acevedo and Jorgensen, 2010; van der Kamp and Mulholland, 2013; Sousa et al., 2017). In QM/MM methods, the chemically active site (i.e., where the chemical reaction takes place or the molecule whose properties are going to be calculated) is treated at the QM level whereas the protein surroundings and the explicit solvent molecules are treated at the MM level. Many possibilities arise depending on the QM and MM methods used and on the QM/MM interface (Senn and Thiel, 2009). Up to now, QM/MM methods have been applied to get insight into different biological issues: (i) enzymatic reaction mechanisms (Friesner and Guallar, 2005; Senn and Thiel, 2007; Acevedo and Jorgensen, 2010), (ii) the calculation of spectroscopic properties (Gascón et al., 2005; Sabin et al., 2010; Gattuso et al., 2017), (iii) the investigation of electronically excited states (Navizet et al., 2010; Sabin et al., 2010; García-Iriepa et al., 2016; Gozem et al., 2017) or (iv) the calculation of pKa values (Jensen et al., 2005; Riccardi et al., 2006). It should be stressed that through QM/MM methods, transition state structures, intermediates and activation energies can be computed, being central for reactivity (Gao et al., 2006; Ramos and Fernandes, 2008; Lonsdale et al., 2012). For this reason, QM/MM methods have become in the last decades an essential tool to get insight into the mechanism of biochemical reactions and hence, to confirm or discard different mechanistic proposals which cannot be elucidated from experimental data.

The multiple strengths of QM/MM methods are that large molecules can be modeled including explicitly the entire system in the calculations, they balance the simulation cost and accuracy and they complement experimental data. On the contrary the main limitation of them is the system setup prior the QM/MM calculations, as there are many possible conformations that the macromolecule can assume, also considering the presence of solvent. However, available experimental data, such as crystallographic structures or physiological measurements, ease the setup preparation.

**Scheme 1:**
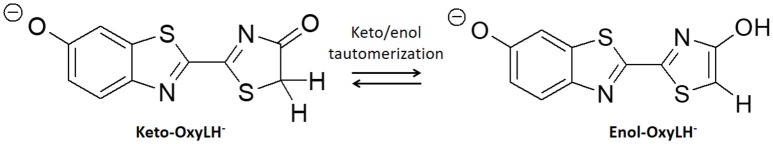
Keto-enol tautomerization of phenolate-oxyluciferin (OxyLH^−^) under study.

In this work we present an application of QM/MM methods to study the bioluminescent system of fireflies, which basis are still not fully understood. In particular, the chemical nature of the emissive specie, so-called oxyluciferin, is still under debate as it can assume six different chemical forms due to triple equilibrium of phenol and/or enol deprotonation together with a keto-enol tautomerization (Hirano et al., 2009; Navizet et al., 2010; Hosseinkhani, 2011). The aim of this work is to elucidate if keto-enol tautomerization (Scheme 1) of phenolate-oxyluciferin (OxyLH^−^) is feasible, both in the ground and in the excited state, inside the active site of the fireflies' luciferase enzyme. For this aim, MD simulations have been performed to sample different possible conformations combined with QM/MM calculations to find the transition states and qualitatively evaluate the energy barriers.

## Methods

### Model setup

**Scheme 2:**
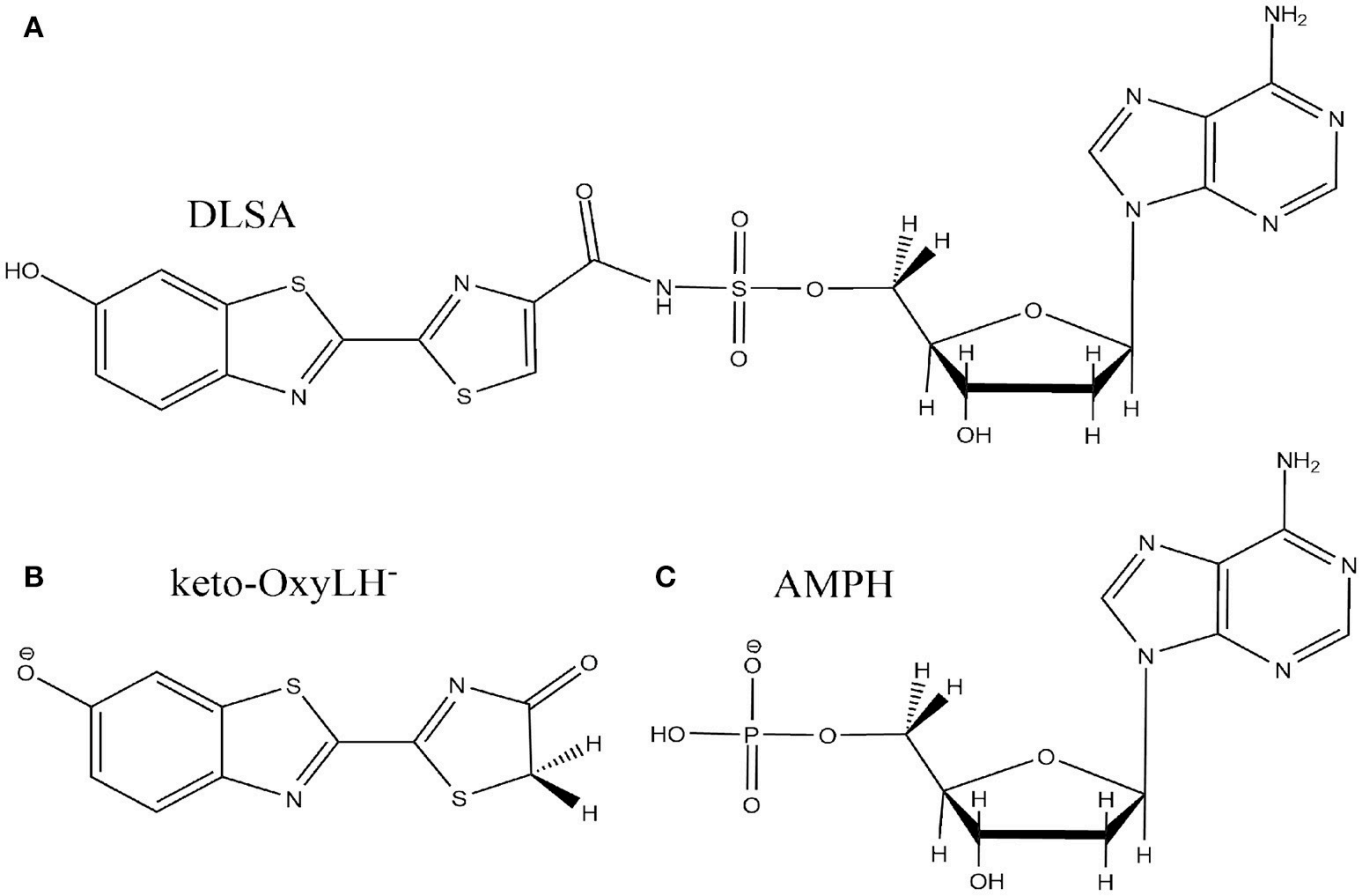
Representation of the DLSA molecule **(A)**, the phenolate-keto oxyluciferin form (keto-OxyLH^−^) **(B)** and AMPH **(C)**.

The crystal structure used in the present publication comes from a north American firefly chemically engineered luciferase, obtained by the group of Prof. Branchini (Branchini et al., 2011; Sundlov et al., 2012) (PDB 4G37). It has been designed to reproduce the conformation of the protein when the dioxygen binds to an intermediate of the bioluminescent reaction. This conformation was obtained thanks to a disulfide bridge between two residues, Isoleucine 108 and Tyrosine 447. Moreover, it contains the 5'-O-[N-(dehydroluciferyl)- sulfamoyl]adenosine (DLSA) (Scheme 2A) substrate.

The missing loops in the crystallographic structure 4G37.pdb downloaded from the RSCB PDB website were added with Disgro program (Tang et al., 2014). Then, the DLSA residue was replaced by protonated adenosine monophosphate AMPH, charged −1, and the phenolate-keto form of oxyluciferin, keto-OxyLH^−^ (Schemes 2B,C). The two ${\text{SO}}_{4}^{2-}$ groups and the disulfide bridging residue were removed. The two ${\text{SO}}_{4}^{2-}$ groups are used as precipitant for the protein (and are not key to model the protein), and were deleted. The S-S cross-linked molecule has also been removed because no accurate parameters are available. No structural changes between the domains of the luciferase were observed during the MD. During the simulations, the protein conformation remains similar to the one of the crystallographic structure. Finally, and in order to neutralize the system, we protonated some histidine residues using the H++ program (Anandakrishnan et al., 2012), keeping unchanged the ones close to the protein active site. In this case, the following histidines were protonated (76, 171, 310, 332, 419, 461 and 489), each one yielding a +1 charge.

### MD simulations

Classical dynamics simulations were made with Amber14 program (Case et al., 2014). The AMBER99ff force field was used to model the residues of the protein. The parameters used for both OxyLH^−^ forms (keto or enol) and AMPH were designed by our group (Navizet et al., 2010, 2011, 2013). The model was solvated with TIP3P (Jorgensen et al., 1983) water molecules within a cube box, ensuring a solvent shell of at least 15 Å around the solute. The resulting system contained roughly 28,000 water molecules and 90,000 atoms in total. The system was heated from 100 to 300 K in 20 ps. Then, under NPT conditions with T = 300 K and P = 1 atm, 21 molecular dynamics of 10 ns using periodic boundary conditions were performed with a 2 fs time step. During these simulations, pressure and temperature were maintained using the Berendsen algorithm and SHAKE constraints were applied to all bonds involving hydrogen atoms (Ryckaert et al., 1977).

### QM/MM setup

The QM/MM calculations have been carried out using a QM/MM coupling scheme (Ferré and Ángyán, 2002) between Gaussian09 (G09 D.01) (Frisch et al., 2010) and Tinker (Ponder, 2004). In particular, the interaction between the QM charge density (electrons and nuclei) and the external electrostatic potential of the MM part was computed by the electrostatic potential fitted (ESPF) method (Ferré and Ángyán, 2002). The microiterations technique (Melaccio et al., 2011) was used to converge the MM subsystem geometry for every QM minimization step. The QM part is composed of the OxyLH^−^, either in the keto or in the enol form, the AMPH and the water molecule n°559 (hereafter named Wat1), placed between the oxyluciferin and AMPH. The rest of the system, that is the protein and the water molecules, are included in the MM part.

QM/MM calculations were used for searching of the transition states (TS) in the protein, defining energetic profiles bridging the TS to the keto and enol forms, and calculating the electronic transitions between the first singlet excited state (S1) and the ground state (GS). For TS and energetic profiles search, unrestricted with broken-symmetry and restricted DFT and TD-DFT calculations were performed using the M06-2X functional (Zhao and Truhlar, 2008) which includes a dispersive term and provides accurate results when studying chemical reactions (Chéron et al., 2012). Unrestricted and restricted calculations gave similar results in terms of energy and geometries, of about 0.12 eV for the transition state (TS), and about 0.15 eV for energetics profiles. Therefore restricted results are presented in this publication. TD-DFT calculations were performed using 3 roots. The emission energy (T_e_) between the first singlet excited state (S1) and the ground state (GS), from geometries obtained in the S1 state, were computed using both the M06-2X and the B3LYP (Stephens et al., 1994) functionals. The B3LYP functional is known to give emission energy values close to experiment for fireflies (Berraud-Pache and Navizet, 2016). Finally, the basis set used is the 6-311G(2d,p) as used in previous publications (Laurent et al., 2015; Berraud-Pache and Navizet, 2016).

### Transition state (TS) search and energetic profiles

**Scheme 3:**
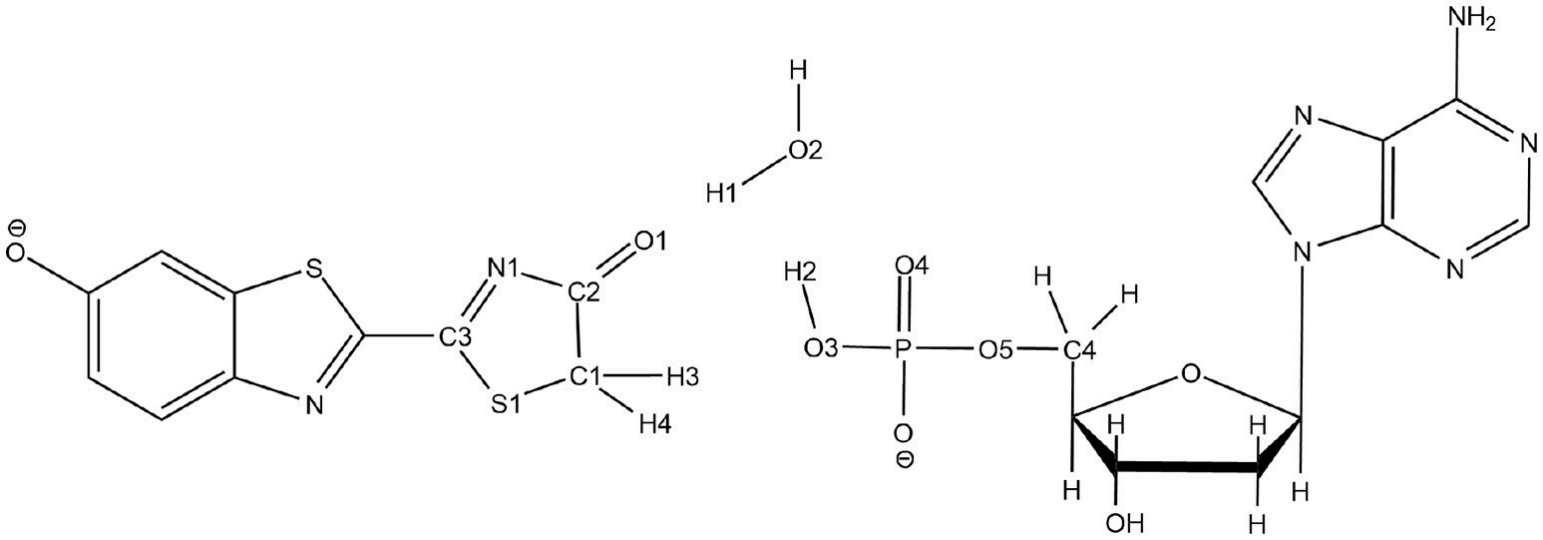
Representation of the QM part extracted from the snapshot and atoms numbering of the relevant atoms.

Once we have selected the MD snapshot for the QM/MM calculations, we can study the keto-enol tautomerization mechanism. Oxyluciferin, AMPH and Wat1 are included in the QM part (Scheme 3) whereas the protein and the other water molecules are treated at the MM level. To study the desired reaction, first the TS connecting the keto and enol forms of oxyluciferin has to be located. However, searching the TS structure inside the protein by QM/MM methods is a complicated task due to both the complexity of the system and the non-availability of certain optimization algorithms [e.g., the Synchronous Transit-Guided Quasi-Newton (STQN) algorithm (Peng and Schlegel, 1993)] in G09/Tinker.

To solve this issue, the QM part selected for QM/MM calculations has been extracted (Scheme 3) and then we have first computed the TS in the GS, without including the protein surrounding but considering water as an implicit solvent by a Polarizable Continuum Model (PCM) (Cancès et al., 1997; Mennucci et al., 1997) of water. Then, the STQN algorithm is used, for which both the keto and the enol structures have to be provided. In our case, the keto form is the one extracted from the selected MD snapshot whereas the enol one is built based on the keto form, just by moving manually H1 to O1, H2 to O2, and H3 to O3 (see Scheme 3 for atoms numbering).

To refine the TS structure obtained with the STQN algorithm, a Berny optimization has been performed. During the Berny optimization, all the non-involved atoms in the tautomerization reaction are blocked (see Supplementary Figure 1). In detail, it corresponds to the benzothiazole cycle of OxyLH^−^, the ribose and the adenosine part of AMPH. A frequency calculation was then performed to validate the found structure as TS, named ${\text{TS}}_{\text{GS}}^{\text{PCM}}$ (see Note 1 in Supplementary Material). Then, the TS in the first excited state, named ${\text{TS}}_{\text{S}1}^{\text{PCM}}$ was computed while considering 3 excited electronics states, starting from the ${\text{TS}}_{\text{GS}}^{\text{PCM}}$ structure.

The keto-enol tautomerization was then studied inside the protein using QM/MM calculations. ${\text{TS}}_{\text{GS}}^{\text{PCM}}$ structure was put in the MD snapshot to replace the QM part coordinates. Then, a Berny optimization has been performed to obtain the transition state in the ground state in the protein, ${\text{TS}}_{\text{GS}}^{\text{prot}}$. Then search of TS in the first excited state in protein starting from ${\text{TS}}_{\text{GS}}^{\text{prot}}$ was performed to obtain ${\text{TS}}_{\text{S}1}^{\text{prot}}$.

To further validate the TS structures, intrinsic reaction coordinate (IRC) calculations have been performed finding that these structures actually connect the keto and the enol forms of oxyluciferin. The IRC has been done for both PCM and QM/MM calculations using the algorithm local quadratic approximation (LQA) (Page and McIver, 1988; Page et al., 1990) and a step of 0.01 Bohr (see Note 2 in Supplementary Material and Supplementary Figure 2).

In the IRC graphs shown in the rest of the publication, the point at the reaction coordinate (RC) equal 0 corresponds to the energy of the relaxed TS structure. Indeed, because some of the atoms are frozen during the Berny optimization, the first step of the IRC shows a big decrease of the energy corresponding to the removing of the constraints of all the atoms of the QM part. However, no geometrical differences are observed between the TS geometry and the relaxed one.

We have also performed a QM/MM dihedral scan of the phosphate group of the AMPH substrate inside the protein at the GS to link the keto-OxyLH^−^ structure form the MD and the one obtained after the IRC search. This scan has been performed along the O4-P-O5-C4 atoms in GS using 10 steps of −10° while the ribose, the adenosine and the OxyLH^−^ were kept frozen.

## Results and discussion

In the present paper, we aim to study the keto-enol tautomerization of oxyluciferin inside the luciferase protein both in the ground state (GS) and in the first singlet excited state (S1). In fireflies, it is well accepted that the final product of the biochemical reaction corresponds to the keto form of oxyluciferin (Liu et al., 2009). However, some experimental measurements (Naumov et al., 2009; Snellenburg et al., 2016) have deciphered the presence of the enol form inside the protein after the bioluminescence emission. The keto-enol tautomerization of oxyluciferin takes place inside the protein. By computing both the GS and S1 energetic profiles of the tautomerization reaction, we can also provide insights to explain if the reaction takes place before or after the emission. The first step is to describe correctly the proteic environment (this has been done by constructing some models from classical molecular dynamics (MD) simulations). We then have to find the geometry of the TSs joining the keto and the enol forms of the oxyluciferin in the proteic environment. As the search of TSs inside the protein, using QM/MM calculations, is complicated we have initially pictured the TSs using a simpler environment, implicit solvation with PCM. Once TSs were located inside the protein, energetic profiles connecting the keto and enol forms were computed.

### Classical MD simulation

First, the system was built starting from the 4G37 luciferase structure as detailed in the Methods section. In this case, the phenolate keto form of oxyluciferin and the AMPH were selected for study.

As demonstrated in previous studies (Navizet et al., 2010; Berraud-Pache and Navizet, 2016), the use of MD simulations is mandatory to investigate the luciferase-oxyluciferin system. MD simulations permit to equilibrate the system, especially important when water molecules are near or in the enzymatic cavity and could lead to a hydrogen-bond network between the substrates and residues of the protein. 21 MD simulations each lasting 10 ns, were performed and thereafter numbered from MD1 to MD21. In all the simulations, we have observed the displacement of water molecules inside the cavity of luciferase. Particularly, in 5 out of the 21 MD simulations, a water molecule (thereafter called Wat1) positions between the keto moiety of oxyluciferin and AMPH. This conformation is stable along the simulation time as a hydrogen-bond network is created involving Wat1, oxyluciferin, AMPH and other protein residues, like LYS 443. By analyzing the MD simulations, we have observed that Wat1 comes close to oxyluciferin at different simulation times depending on the dynamic. For example, Wat1 comes close to oxyluciferin at around 1 ns in MD1, whereas it takes more than 6 ns to reach this position in MD17.

The position of Wat1 suggests that this water molecule could be involved in the keto-enol tautomerization mechanism of oxyluciferin inside the protein. This way, the tautomerization could take place by the displacement of 3 protons: one from oxyluciferin, one from Wat1 and one from AMPH. We therefore decided to study the feasibility of this mechanism both in the GS and in S1 inside the protein.

**Figure 1:**
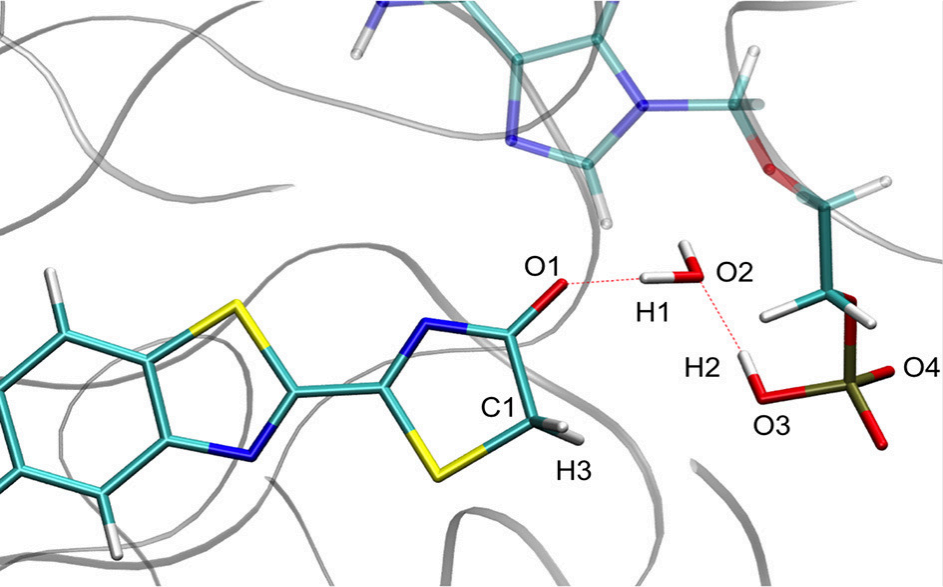
Representation of the MD snapshot selected as starting structure for QM/MM calculations. The oxyluciferin is represented in its keto form.

For calculation purposes, we selected one representative snapshot corresponding to the lowest structure in energy found along the MD (snapshot taken at 3.708 ns from MD1). In this snapshot a water molecule Wat1 was found between oxyluciferin and AMPH. By analyzing in more detail the structure of this snapshot (Figure 1), we observe that the nearby residues do not share any hydrogen-bond with the substrates or Wat1. Only the hydrogen-bond network between oxyluciferin, AMPH and Wat1 is present. Thus, the choice of this snapshot and hence of this hydrogen-bond network was motivated to simplify the system, preventing the inclusion of more residues in the QM part, which would have increased the computational cost.

### Description of the TS in implicit solvent

The optimized TS structure in the GS computed in PCM (${\text{TS}}_{\text{GS}}^{\text{PCM}}$) corresponds to a 3 protons transfer reaction between 4 centers: O1, O2, O3 and C1 (see Supplementary Figure 3). Moreover, the ${\text{TS}}_{\text{GS}}^{\text{PCM}}$ is structurally more similar to the keto form of oxyluciferin than to the enol form. For instance, H1 is closer to O2 of Wat1 (0.98 Å) than to O1 of oxyluciferin and H2 is closer to O3 of AMPH (1.03 Å) than to O2 of Wat1.

However, for the TS optimized in S1 in PCM (${\text{TS}}_{\text{S}1}^{\text{PCM}}$), the position of O4 leads to a 5 centers TS, including O1, O2, O3, O4, and C1. It allows a bigger flexibility for the atoms and for the proton transfer. In ${\text{TS}}_{\text{S}1}^{\text{PCM}}$ (see Supplementary Figure 4), H1 is closer to O1 (1.01 Å) and H3 is still located between C1 and O3. The notable difference with the ${\text{TS}}_{\text{GS}}^{\text{PCM}}$ is the presence of O4, another oxygen of the AMPH substrate, which is involved in the tautomerization reaction. Here the H2 is not located between O2 and O3 as in ${\text{TS}}_{\text{GS}}^{\text{PCM}}$ but between O2 and O4. During the optimization of ${\text{TS}}_{\text{S}1}^{\text{PCM}}$, the phosphate group of the AMPH is rotated about 30°, bringing the O4 close to the water molecule. Some details about the IRC calculations with implicit solvent can be found in note 3 and Supplementary Figure 5.

In conclusion, when computing the TS in PCM both in the GS and in S1 (${\text{TS}}_{\text{GS}}^{\text{PCM}}$ and ${\text{TS}}_{\text{S}1}^{\text{PCM}}$), two different TS geometries are obtained. The tautomerization mechanism concerning ${\text{TS}}_{\text{GS}}^{\text{PCM}}$ is a 4 centers reaction while for ${\text{TS}}_{\text{S}1}^{\text{PCM}}$, 5 centers are involved. This study shows that a TS connecting the keto and enol forms of oxyluciferin can be obtained with PCM. The computed geometry of ${\text{TS}}_{\text{GS}}^{\text{PCM}}$ with PCM has served as starting guess for the calculation of the TS in GS inside the protein at the QM/MM level, which is the main goal of the present study.

### Keto-enol tautomerization inside the protein

We have decided to use ${\text{TS}}_{\text{GS}}^{\text{PCM}}$, a 4 centers TS, as starting structure for the QM/MM calculations inside the protein. Indeed, in all the computed MD Wat1 adopts a position that suggests a 4 centers TS, as seen in Scheme 3 and Figure 1.

The TS optimization done inside the protein in the GS gives some unexpected results. First, two different TS geometries can be obtained, both corresponding to the keto-enol tautomerization. In the first one, the position of the 3 protons suggests that the TS is derived from the enol tautomer of the oxyluciferin. It is thus named ${\text{TS}}_{\text{GS\_enol}}^{\text{prot}}$. In the same way, the second TS, named ${\text{TS}}_{\text{GS\_keto}}^{\text{prot}}$, shows protons close to the position adopted in the keto form. It should be remarked here that the ${\text{TS}}_{\text{GS\_enol}}^{\text{prot}}$ lies *ca*. 34 kcal/mol above in energy compared to ${\text{TS}}_{\text{GS\_keto}}^{\text{prot}}$ in the GS.

**Scheme 4:**
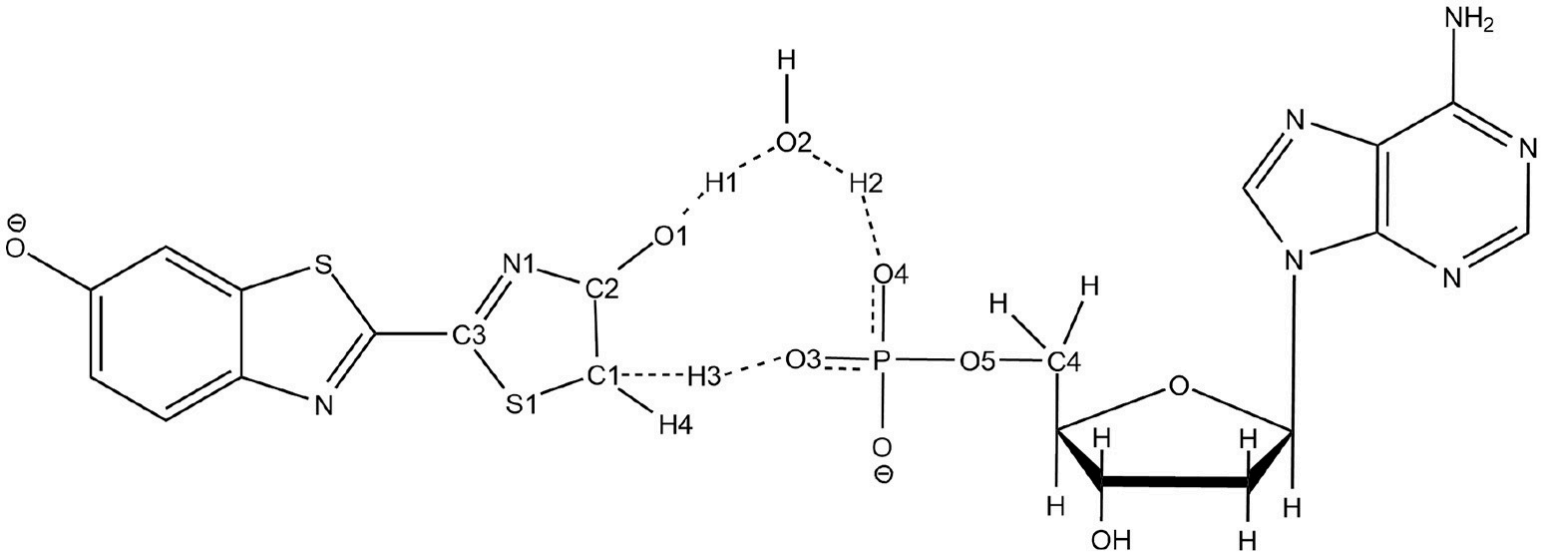
Representation of the 5 centers mechanism for keto-enol tautomerization inside the protein.

The second unexpected result concerns the geometries of all computed TSs. When using QM/MM calculations inside the protein, only a 5 centers tautomerization is observed in the GS even when starting with the 4 centers ${\text{TS}}_{\text{GS}}^{\text{PCM}}$. Within the protein active site, the substrates prefer to fill more space when performing the keto-enol tautomerization. All other attempts to compute a 4 centers TS in the GS failed, giving in all cases a 5 centers one (see Scheme 4).

Finally, the TSs in S1 have been located starting from both the structure of ${\text{TS}}_{\text{GS\_keto}}^{\text{prot}}$ and of ${\text{TS}}_{\text{GS\_enol}}^{\text{prot}}$. As in the GS, two different TS structures have been found in S1, one structurally closer to the keto form, ${\text{TS}}_{\text{S}1\text{\_keto}}^{\text{prot}}$, and the other one closer to the enol form, ${\text{TS}}_{\text{S}1\text{\_enol}}^{\text{prot}}$. The ${\text{TS}}_{\text{S}1\text{\_keto}}^{\text{prot}}$ is also lower in energy compared to ${\text{TS}}_{\text{S}1\text{\_enol}}^{\text{prot}}$. These differences are detailed in the Keto-enol tautomerization subsection.

### Geometrical description of the TSs

**Figure 2:**
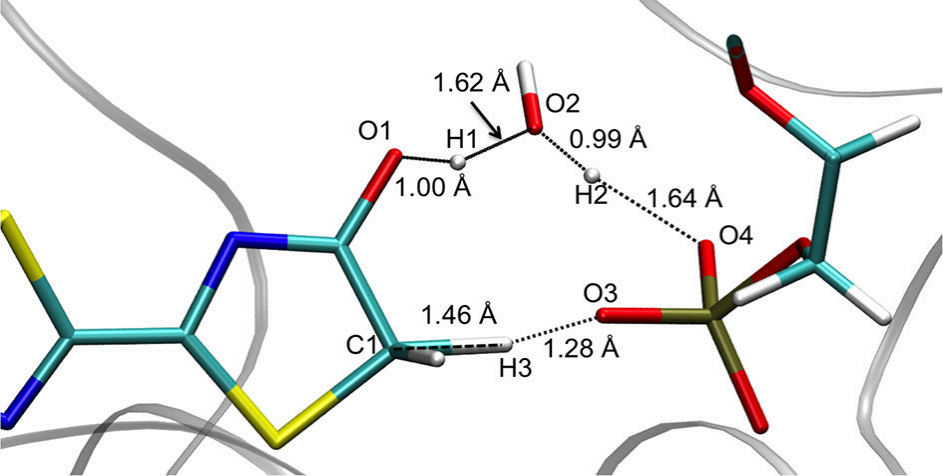
Structure of ${\text{TS}}_{\text{S}1\text{\_enol}}^{\text{prot}}$ computed at the QM/MM level.

The ${\text{TSs}}_{\text{enol}}^{\text{prot}}$ structures obtained in both GS and S1 are quite similar. They can be described by the transfer of 3 protons (H1, H2, and H3) between 5 centers (O1, O2, O3, O4, and C1) (see Figure 2). In particular, H1 is placed between O1 (oxyluciferin) and O2 (Wat1) but it is closer to O1 (1.00 Å) and so, resembles the enol form. Moreover, H2 is located close to O2 (Wat1) (0.99 Å) while H3 is situated between C1 and O3 (d_H3−C1_ = 1.46 Å and d_H3−O3_ = 1.28 Å).

**Figure 3:**
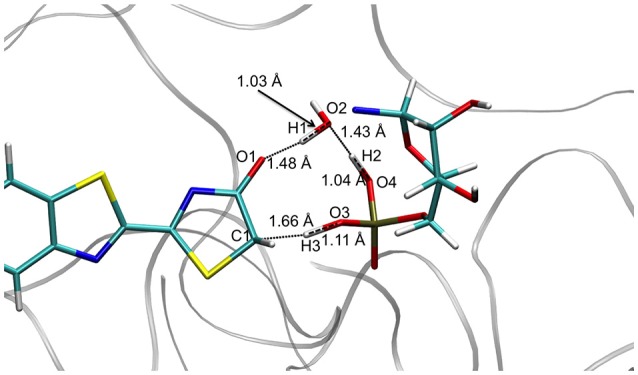
Structure of ${\text{TS}}_{\text{S}1\text{\_keto}}^{\text{prot}}$ computed at the QM/MM level.

Concerning the ${\text{TSs}}_{\text{keto}}^{\text{prot}}$, the structures computed in the GS and in S1 are also quite similar, corresponding to the transfer of 3 protons involving 5 centers (see Figure 3). However, in these cases H1 is closer to O2 (Wat1) (1.03 Å) than to O1 (oxyluciferin) (1.48 Å). Moreover, H2 is located close to O4 (AMPH) (1.04 Å) and H3 is nearly bonded to O3 (AMPH) (1.11 Å).

### Analysis of the energetic profiles of the two TSs

**Figure 4:**
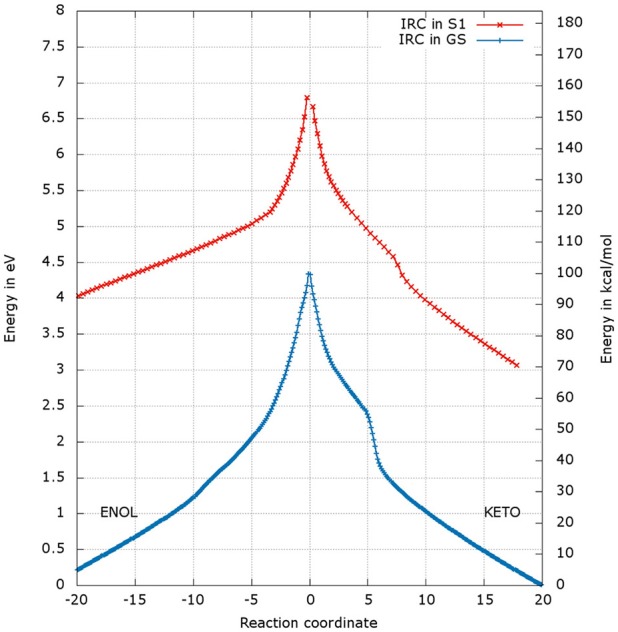
Energetic profile of the keto-enol tautomerization starting from ${\text{TS}}_{\text{GS\_enol}}^{\text{prot}}$ and ${\text{TS}}_{\text{S}1\text{\_enol}}^{\text{prot}}$ at the reaction coordinate RC = 0. Superposition of GS in blue and S1 profiles in red. Positive RC values lead to the keto form while the negative ones to the enol form. The point at 0 eV corresponds to the lowest point of the energy profile that is to the keto form in the GS. The graph is a superposition of two graphs: reaction coordinates of the IRCs in the GS and in S1 are not the same.

**Figure 5:**
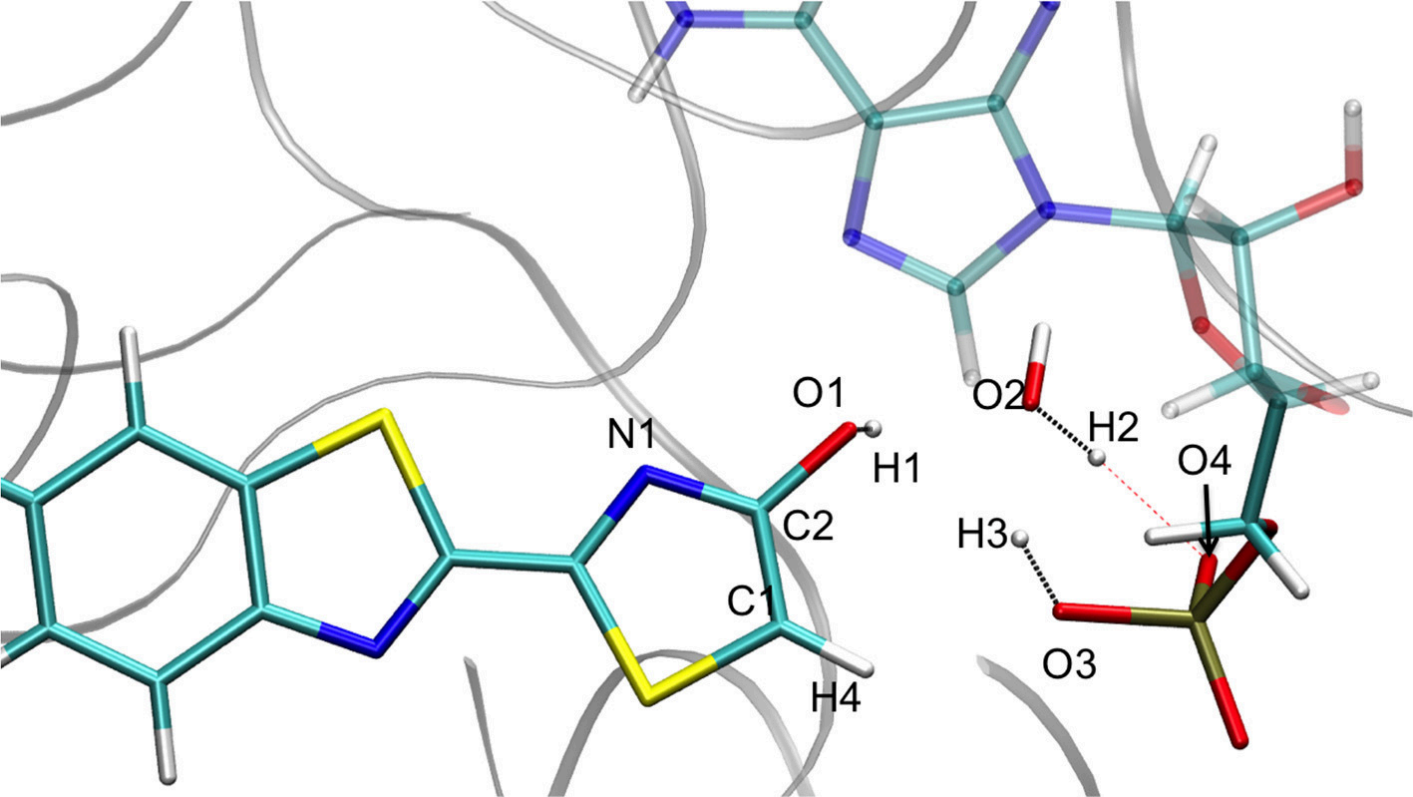
Representation of the enol tautomer obtained from the IRC calculation starting from ${\text{TS}}_{\text{S}1\text{\_enol}}^{\text{prot}}$ in S1 inside the protein. The structure is similar to the one starting from the ${\text{TS}}_{\text{S}1\text{\_keto}}^{\text{prot}}$.

After the optimization of the TSs, we computed the IRCs to check if they really connect the keto and enol forms of oxyluciferin both in the GS and in S1. The results presented in Figures 4, 5 correspond to a superposition of the IRC computed in the GS and in S1 for a better comparison. However, reader has to have in mind that the reaction coordinates of the IRCs in the GS and in S1 are not the same. Moreover, the TS structure has been defined as the 0 value of the reaction coordinate. When moving to the right toward positive values of the reaction coordinate, the keto tautomer is formed. Similarly, negative values of the reaction coordinate correspond to the formation of the enol form. The energy of the keto tautomer in the GS was considered as the reference.

### Energetic profile of the TS_enol

The shapes of the energetic profiles starting from ${\text{TS}}_{\text{GS\_enol}}^{\text{prot}}$ and ${\text{TS}}_{\text{S}1\text{\_enol}}^{\text{prot}}$ in the GS and in S1, respectively, are similar (see Figure 4). Moreover, it is observed that, the keto form is more stable than the enol form of about 5 kcal/mol in the GS and 11 kcal/mol in S1.

When following the energetic curve in the direction of the enol tautomer (i.e., negative values of reaction coordinate RC), we observed a steep decrease of the energy followed by a smaller slope. The steep gradient corresponds mainly to the movement of the proton H3, as the protons H1 and H2 are already in the final position of the enol form in the ${\text{TS}}_{\text{enol}}^{\text{prot}}$, both in the GS and in S1. Thus, at about RC equal −4, the enol tautomer is already formed. From this point, the system stabilization is due to a rearrangement of oxyluciferin and AMPH leading to the final enol structure (Figure 5). Finally, the energy barrier for tautomerization starting from the enol form found is 100 kcal/mol for the GS and 66 kcal/mol for the S1.

**Figure 6:**
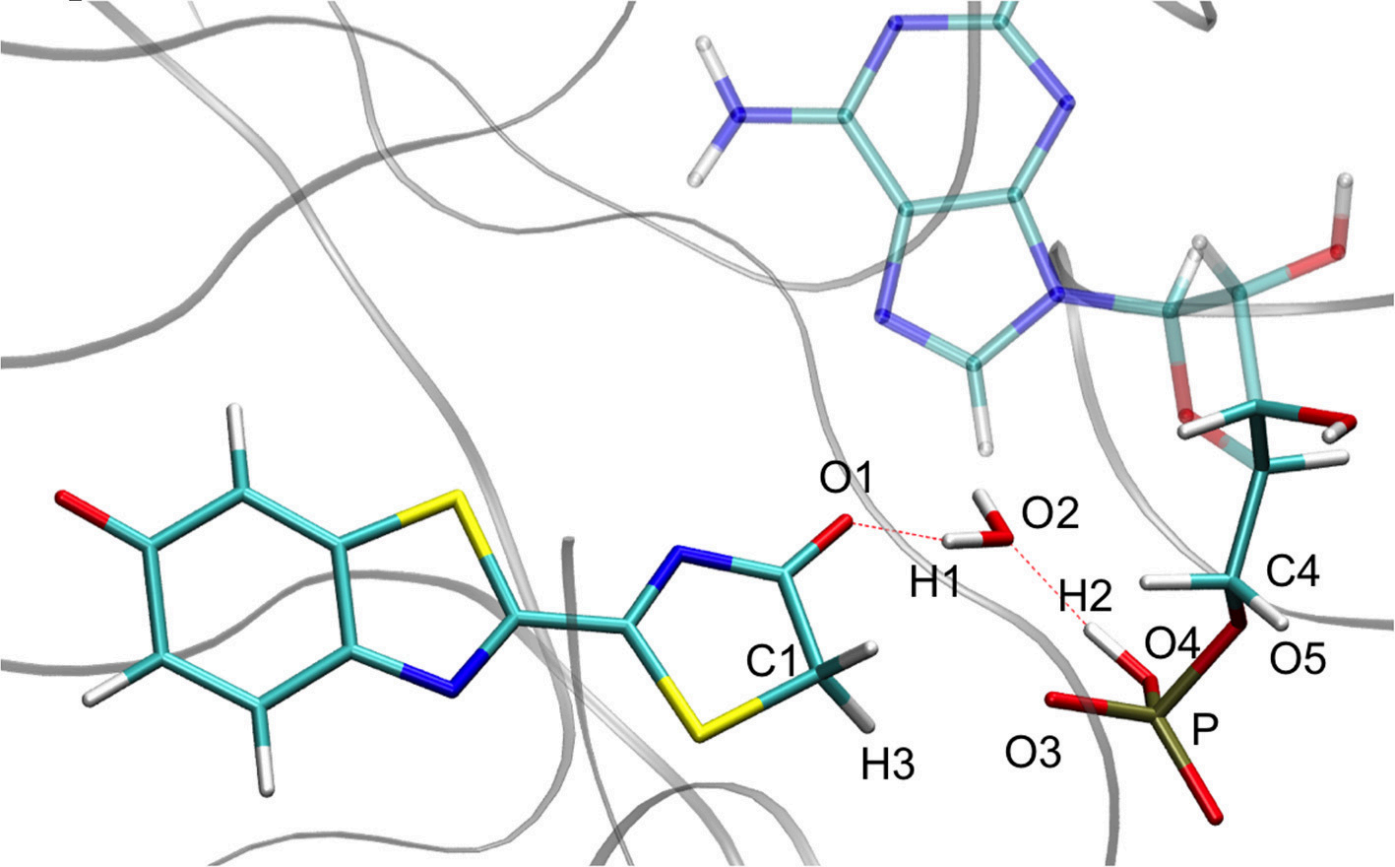
Representation of the keto tautomer obtained from the IRC calculation starting from ${\text{TS}}_{\text{S}1\text{\_enol}}^{\text{prot}}$ in S1 inside the protein. The structure is similar to the one starting from the ${\text{TS}}_{\text{S}1\text{\_keto}}^{\text{prot}}$.

On the side of the curve between the TS and the keto tautomer (i.e., positive values of reaction coordinate), the profile can also be divided into two steps. In the GS curve, between RC 0 and RC 5, the proton H3 is moving in direction of C1. The shoulder at RC equal 5 corresponds to the break of the bonds O1-H1 and O2-H2. From this point, the system minimization can be described by the movement of these two protons H1 and H2. The final structure is represented in Figure 6. The energetic barrier for tautomerization starting from the keto form is of 105 kcal/mol for the GS and 77 kcal/mol for the S1.

Finally, in order to verify that the structures obtained at the end of the IRC calculations correspond to the keto and enol tautomers (Figures 5, 6), we have computed their electronic transition energies (T_e_) between S1 and the GS (respectively ${\text{T}}_{\text{e\_keto\_IRC}}^{\text{prot}}$ and ${\text{T}}_{\text{e\_enol\_IRC}}^{\text{prot}}$). The electronic transition between S0 and S1 corresponds to a π-π^*^ transition with a small charge transfer between the benzothiazole ring and the thiazolone ring for both tautomers. Then, the computed energies have been compared to reference values (${\text{T}}_{\text{e\_keto\_ref}}^{\text{prot}}$ and ${\text{T}}_{\text{e\_enol\_ref}}^{\text{prot}}$) obtained directly from the MD snapshots of these oxyluciferin forms (see Note 4 in Supplementary Material). T_e_ have been computed with both, the M06-2X and the B3LYP functionals, the last one giving emission energy values closer to experiment (Berraud-Pache and Navizet, 2016) (see Table 1). The computed electronic transition energies of the keto and enol forms obtained by the IRC show good agreement with reference values. The difference of energy is less than 0.1 eV, which corresponds to the DFT level of incertitude.

**Table 1:** Electronic transition energies between S1 and GS (T_e_) in the protein at the TD-DFT/MM level for the resulting structures of the IRC, starting with ${\text{TS}}_{\text{S}1\text{\_enol}}^{\text{prot}}$.

	**Structure obtained in S1**	**M06-2X T_e_ eV (nm)**	**B3LYP T_e_ eV (nm)**
keto-OxyLH^−^	Last point of the profile:
	${\text{T}}_{\text{e\_keto\_IRC}}^{\text{prot}}$	2.65 (472)	2.34 (529)
	${\text{T}}_{\text{e\_keto\_ref}}^{\text{prot}}$	2.55 (486)^a^	2.24 (555)^a^
enol-OxyLH^−^	Last point of the profile:
	${\text{T}}_{\text{e\_enol\_IRC}}^{\text{prot}}$	2.83 (439)	2.52 (492)
	${\text{T}}_{\text{e\_enol\_ref}}^{\text{prot}}$	2.86 (434)^a^	2.47 (502)^a^

a *Data in SI*.

### Energetic profile of the TS_keto

**Figure 7:**
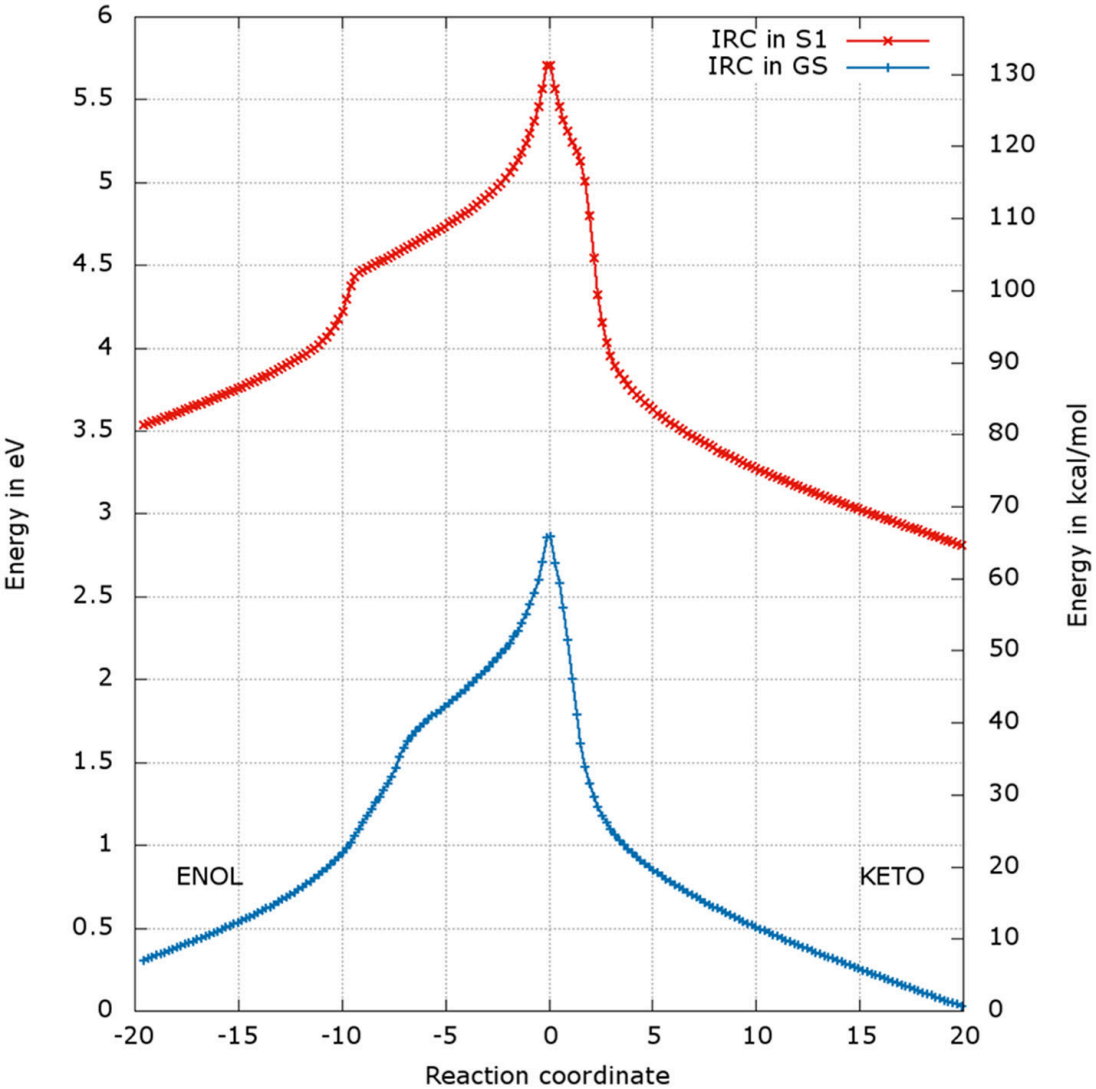
Energetic profile of the keto-enol tautomerization starting from the ${\text{TS}}_{\text{GS\_keto}}^{\text{prot}}$ and ${\text{TS}}_{\text{S}1\text{\_keto}}^{\text{prot}}$ at the reaction coordinate RC = 0. Superposition of GS in blue and S1 profiles in red. Positive RC values lead to the keto form while negative ones to the enol form. The point at 0 eV corresponds to the lowest point of the energy profile that is to the keto form in the GS. The graph is a superposition of two graphs: reaction coordinates of the IRCs in the GS and in S1 are not the same.

The energetic profiles found in the GS and in S1 starting from ${\text{TS}}_{\text{GS\_keto}}^{\text{prot}}$ and ${\text{TS}}_{\text{S}1\text{\_keto}}^{\text{prot}}$, respectively, are similar (see Figure 7). The keto tautomer is again more stable than the enol one, of about 7 kcal/mol in the GS and 15 kcal/mol in S1. Concerning the formation of the enol form (negative values of RC), H3 moves first, corresponding to the shoulder observed at RC −7 in the GS and RC −9 in S1. Then, the bonds H1-O2 and H2-O3 break and the two protons H1 and H2 move toward O1 and O2 respectively, leading to the enol tautomer. The computed energetic barriers for the tautomerization starting from the enol form are 58 kcal/mol in the GS and 51 kcal/mol in S1.

For the formation of the keto form, positive RC values, a steep gradient is observed that corresponds to the movement of H3. The calculated energetic barriers from the keto form to the TS are 65 kcal/mol in the GS and 67 kcal/mol in S1.

Finally, the T_e_ have also been computed for the obtained structures reached by the IRC (see Figures 5, 6), starting from ${\text{TS}}_{\text{S}1\text{\_keto}}^{\text{prot}}$ (see Table 2). The electronic transition between S0 and S1 corresponds to a π-π^*^ transition with a small charge transfer between the benzothiazole ring and the thiazolone ring for both tautomers. The computed values are again in good agreement with previous results (see Note 4 in Supplementary Material), being the energy differences between 0.09 and 0.13 eV.

**Table 2:** Electronic transition energies between S1 and GS (T_e_) in the protein at the TD-DFT/MM level for the resulting structures of the IRC, starting with ${\text{TS}}_{\text{S}1\text{\_keto}}^{\text{prot}}$.

	**Structure obtained in S1**	**M06-2X T_e_ eV (nm)**	**B3LYP T_e_ eV (nm)**
keto-OxyLH^−^	Last point of the profile:		
	${\text{T}}_{\text{e\_keto\_IRC}}^{\text{prot}}$	2.64 (470)	2.36 (526)
	${\text{T}}_{\text{e\_keto\_ref}}^{\text{prot}}$	2.55 (486)^a^	2.24 (555)^a^
enol-OxyLH^−^	Last point of the profile:		
	${\text{T}}_{\text{e\_enol\_IRC}}^{\text{prot}}$	2.73 (451)	2.59 (479)
	${\text{T}}_{\text{e\_enol\_ref}}^{\text{prot}}$	2.86 (434)^a^	2.47 (502)^a^

a *Data in SI*.

### Rotation

**Figure 8:**
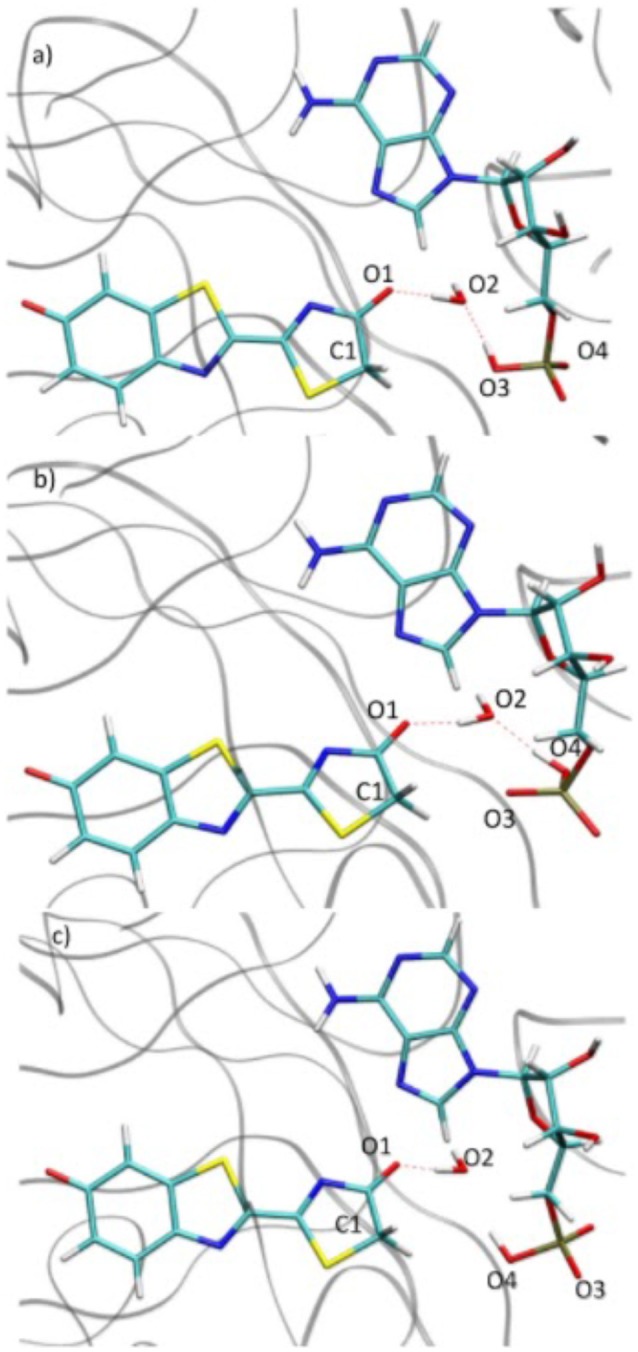
Comparison of the structure extracted from the MD **(a)**, the structure corresponding to the last point of the IRC toward the keto form in GS **(b)** and the structure obtained after the dihedral scan **(c)**.

During the study of the tautomerization inside the protein, we find that the reaction involves 5 centers. Thus, the keto form from the MD snapshot (Figures 1, 8a) does not match with the one obtained by the IRC calculations in the GS starting from both, the ${\text{TS}}_{\text{GS\_ketol}}^{\text{prot}}$ or the ${\text{TS}}_{\text{GS\_enol}}^{\text{prot}}$ (Figures 6, 8b). Indeed, by construction of the AMPH classical structure, the proton H2 is bound to the oxygen O3 in all MD. In the 5 centers reaction inside the protein leading to the keto form, the O4 from the AMPH retrieve the proton H2. The resulting keto system shows therefore a protonated AMPH on its O4 oxygen.

**Figure 9:**
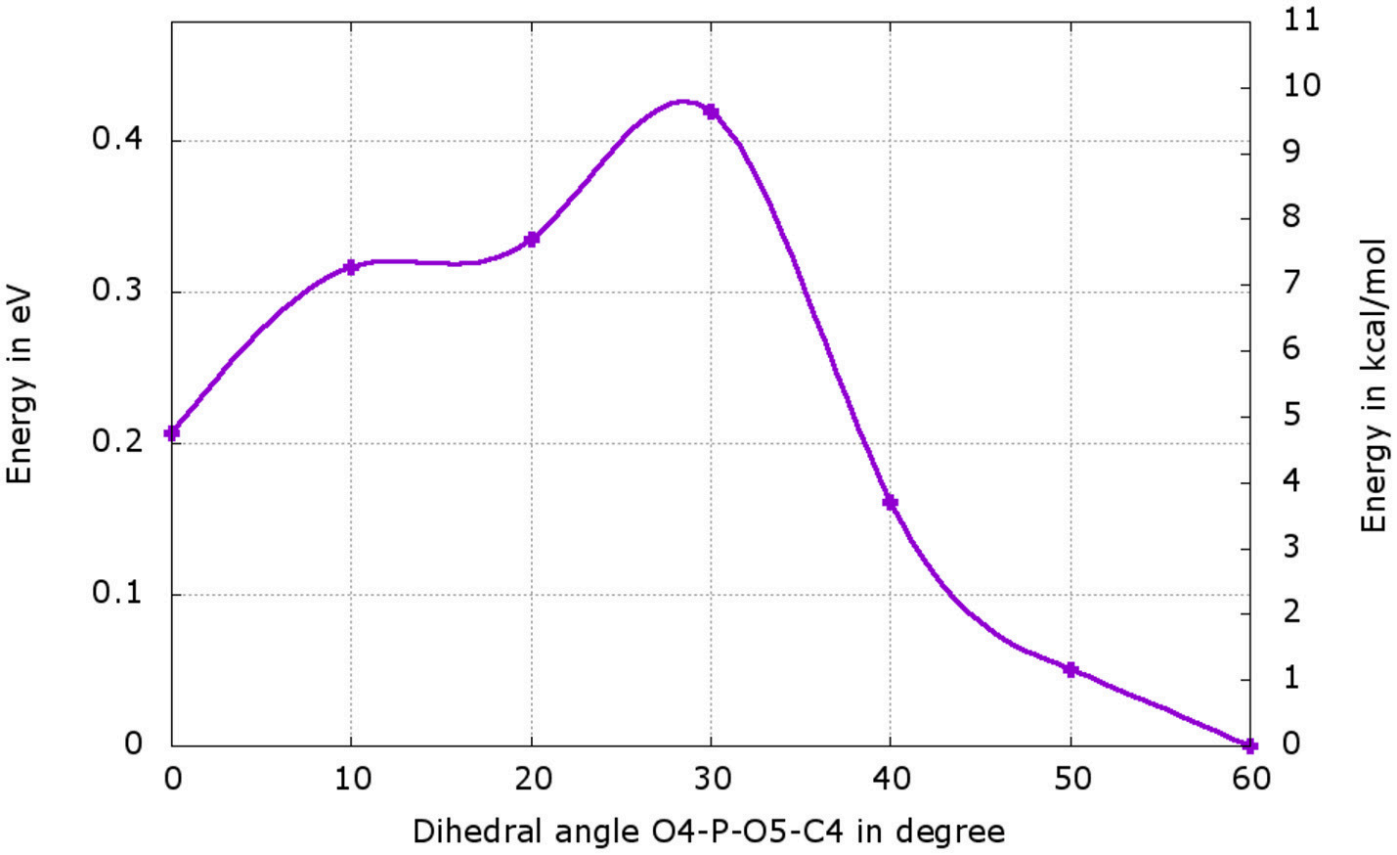
Energetic profile of the scan around the O4-P-O5-C4 dihedral. The structure with dihedral at 0° corresponds to the last point of the IRC starting from both, the ${\text{TS}}_{\text{GS\_ketol}}^{\text{prot}}$ or the ${\text{TS}}_{\text{GS\_enol}}^{\text{prot}}$, (Figure 8b). The point with dihedral at 60° (Figure 8c) corresponds to a structure similar to the starting structure extracted from the MD (Figure 8a).

We have performed a dihedral scan to see the energy needed to allow the rotation of the phosphate group. We have selected the last point from the IRC calculation and performed a scan in GS around the dihedral O4-P-O5-C4 inside the protein. The same scan at the S1 level would be much longer to obtained and we hypothesize that the results would not be very different from the ones in GS. When looking at the energetic profile, the energy reaches a minimum when the hydroxyl group O4-H2 has the same position as O3-H2 observed during the MD (see Figure 8). However, the difference of stability and the rotation barrier between the two structures is about 5 kcal/mol, which is rather small (see Figure 9).

### Keto-enol tautomerization

We have found that using QM/MM calculations different TSs, ${\text{TSs}}_{\text{keto}}^{\text{prot}}$ and ${\text{TSs}}_{\text{enol}}^{\text{prot}}$, both in the GS and in S1, could lead to the keto-enol tautomerization. Besides, both TSs correspond to a 5 centers mechanism, involving two oxygen atoms of the phosphate group of the AMPH. In the ${\text{TSs}}_{\text{keto}}^{\text{prot}}$, the oxyluciferin is close to the keto conformation while it looks more like the enol form in the ${\text{TSs}}_{\text{enol}}^{\text{prot}}$. The energy of the ${\text{TSs}}_{\text{keto}}^{\text{prot}}$ is always lower than the one of ${\text{TSs}}_{\text{enol}}^{\text{prot}}$, both in GS and in S1. The energy difference between the ${\text{TSs}}_{\text{keto}}^{\text{prot}}$ and the ${\text{TSs}}_{\text{enol}}^{\text{prot}}$ is quite high, about 34 kcal/mol in GS and 26 kcal/mol in S1. In all calculations we have done, the keto-OxyLH^−^ is always more stable than the enol form, therefore it is not surprising to find ${\text{TSs}}_{\text{keto}}^{\text{prot}}$ more stable than ${\text{TSs}}_{\text{enol}}^{\text{prot}}$.

This difference of energy also plays a major role in the barriers heights. The ones computed for the ${\text{TSs}}_{\text{keto}}^{\text{prot}}$ are significantly lower than the ones of ${\text{TSs}}_{\text{enol}}^{\text{prot}}$, both in GS and in S1. However, the computed values remain high (the lowest computed one is *ca*. 51 kcal/mol, see Table 3) compared to previous studies of keto-enol tautomerization (*ca*. 40 kcal/mol) (Cucinotta et al., 2006; Alagona and Ghio, 2008).

**Table 3:** Energy barriers in kcal/mol of keto-enol and enol-keto tautomerization paths, in the GS and in S1, through ${\text{TSs}}_{\text{keto}}^{\text{prot}}$ and ${\text{TSs}}_{\text{enol}}^{\text{prot}}$.

	**Through TS**${\;}_{\text{keto}}^{\text{prot}}$		**Through TS**${\;}_{\text{enol}}^{\text{prot}}$	
	**GS**	**S1**	**GS**	**S1**
Keto to enol path	65	67	105	77
Enol to keto path	58	51	100	66

The presence of the ${\text{TSs}}_{\text{keto}}^{\text{prot}}$ and ${\text{TSs}}_{\text{enol}}^{\text{prot}}$ also raises the question of a preferable pathway. Indeed, one possible hypothesis is that, because the ${\text{TSs}}_{\text{keto}}^{\text{prot}}$ structures are closer to the keto-OxyLH^−^ geometry, the tautomerization reaction from the keto tautomer to the enol tautomer might go through the ${\text{TSs}}_{\text{keto}}^{\text{prot}}$. The reverse reaction, enol to keto tautomerization, should also go through the ${\text{TSs}}_{\text{keto}}^{\text{prot}}$ as they are lower in energy but because the s${\text{TS}}_{\text{enol}}^{\text{prot}}$ protons' arrangement is close to the reactant enol, the path through these TSs might also be considered.

From our calculations and with the hypothesis we have taken, the energy barriers calculated in the protein are very high and show that the tautomerization would not be easy inside the protein both in GS and S1. Experimental results show that after the reaction, a mixing keto and enol forms have been detected inside the protein (Naumov et al., 2009). However, other experimental results show that the keto-OxyLH^−^ tautomer is the only bioluminescence emitter in fireflies, as one other recent study also shows (Pirrung et al., 2017). From all these results, we can deduce that the tautomerization is most probably difficult in S1 and, for it to happen in GS after light emission, the protein environment should change (for example, movement of the C-term and rearrangement of the H-bonding network). This is still to be proved with further calculations, especially when using other hypothesis like the protonation state of AMP, using more snapshot from the MD, or using a bigger QM region. Moreover, some other details can be taken into account to refine the model. In the chosen snapshot, the QM part does not exhibit strong interaction with the protein. As we already mentioned before, some water molecules or residues can form hydrogen bonds with the concern atoms of the tautomerization reaction.

The use of QM/MM calculations has provided a better model regarding the keto-enol tautomerization compared to implicit solvent model. The main finding concerns the characterization of the TSs. Inside the protein, the concerted displacement of 3 protons is described. The 5 centers TS geometry shows that the active site of the protein is quite flexible and can sustain a complex chemical reaction.

## Conclusion

In this publication we demonstrate the possibility to explore chemical reactions using QM/MM calculations by the study of keto-enol tautomerization of the emitter of the bioluminescence in fireflies.

Extensive MD calculations show a recurrence of the presence of a water molecule between the oxyluciferin and AMPH, which allows 3 protons transfer during tautomerization. Preliminary QM calculations in PCM are necessary to guess the TSs as models for further calculation at the QM/MM level.

The use of QM/MM calculations to study the chemical reaction unveils some unexpected results. First, the reaction is possible in protein when 5 chemical centers are involved, in contrary to the PCM study, where only a structure compatible with the 4 centers reaction is observed in the GS. Secondly, we have found two different TSs that can carry out the tautomerization reaction. These TSs reflect a tautomer of the emitter, one is similar to the keto form while the other is close to the enol one. In addition, these TSs have distinct energies, the ${\text{TSs}}_{\text{keto}}^{\text{prot}}$ are the most stable ones in both the GS and S1.

The computed barriers are quite high or even impassable. It is thus nowadays complicated to think that the keto-enol tautomerization can take place inside the protein in the excited state before emitting. This gives another proof of the role of the keto-OxyLH^−^ as the main emitter of the bioluminescence.

The results presented here can be improved in different ways. It is possible to take into account several snapshots and improve the general picture of the system. For this purpose, we are presently studying the emission spectra simulation of different analogs of the oxyluciferin using a statistical number of snapshots. Moreover, some residues can have an effect on the barrier height. The residue LYS 443, which is important in fireflies seems a good participant. Other methods can be used and have already prove efficiency, like QM/MM dynamics or meta-dynamics (Cucinotta et al., 2006).

We think that this protocol can be applied to other biological systems, like DNA (Cerón-Carrasco and Jacquemin, 2013), and bring new insights in modeling chemical reactions.

## Author contributions

All authors listed, have made substantial, direct and intellectual contribution to the work, and approved it for publication.

### Conflict of interest statement

The authors declare that the research was conducted in the absence of any commercial or financial relationships that could be construed as a potential conflict of interest.
