# Supplementary material for: Modeling Chemical Reactions by QM/MM Calculations: The Case of the Tautomerization in Fireflies Bioluminescent Systems
Source: Front Chem. 2018 Apr 17;6:116. doi: 10.3389/fchem.2018.00116 (PMC5913368; doi:10.3389/fchem.2018.00116)
Supplement: Supplementary file 1 [file DataSheet1.PDF]

Supplementary Material

Modelling chemical reactions by QM/MM calculations: the case of the tautomerization in fireflies bioluminescent systems.

Romain Berraud-Pache[1], Cristina Garcia-Iriepa[1], Isabelle Navizet[*1]

*** Correspondence:** Corresponding Author: isabelle.navizet@u-pem.fr

1 Supplementary Figures

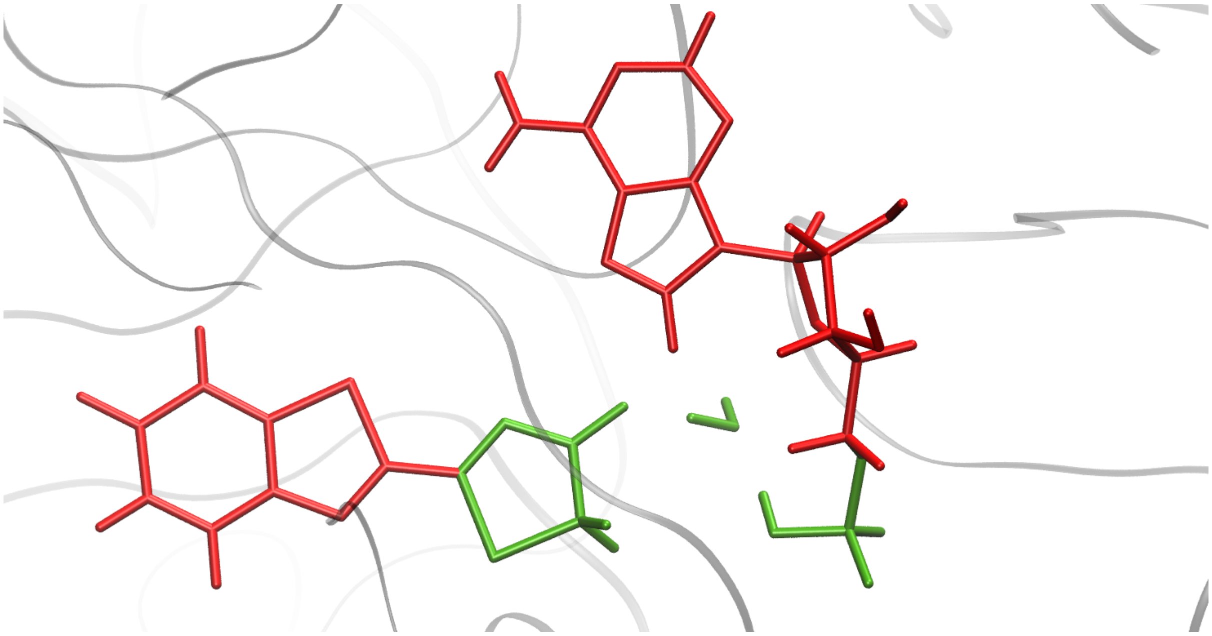

Supplementary Figure 1. Graphical representation of the constrained atoms during the Berny optimization. In green, the atoms are not constrained while in red they are frozen.

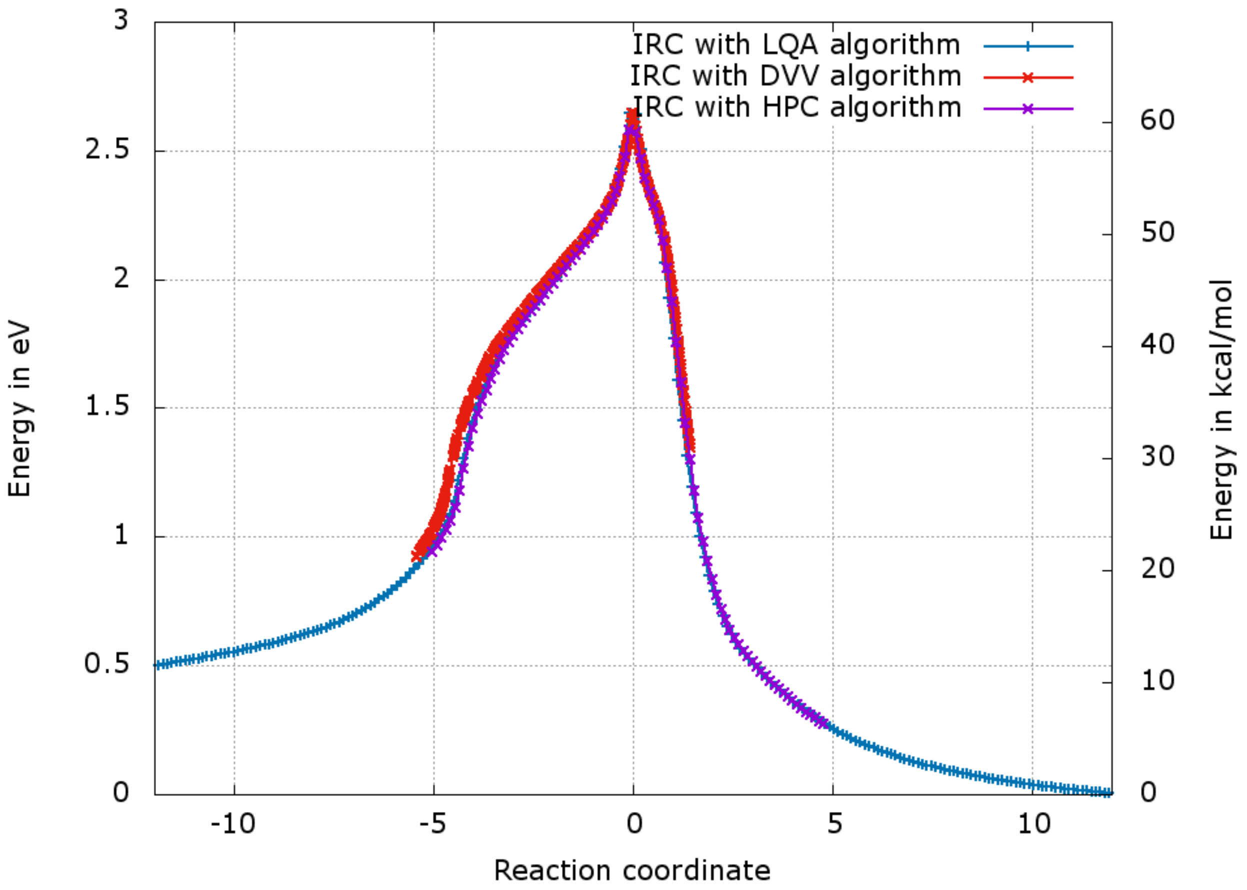

Supplementary Figure 2. Energetic profile of the keto-enol tautomerization starting from TS_{GS}^{PCM} at the reaction coordinate RC = 0. Comparison between 3 IRC algorithms, LQA (lines), DVV (cross) and HPC (points). Positive RC leads to the keto form while negative one to the enol form. The point at 0 eV corresponds to the lowest point of the energy profile that corresponds to the keto form in the GS.

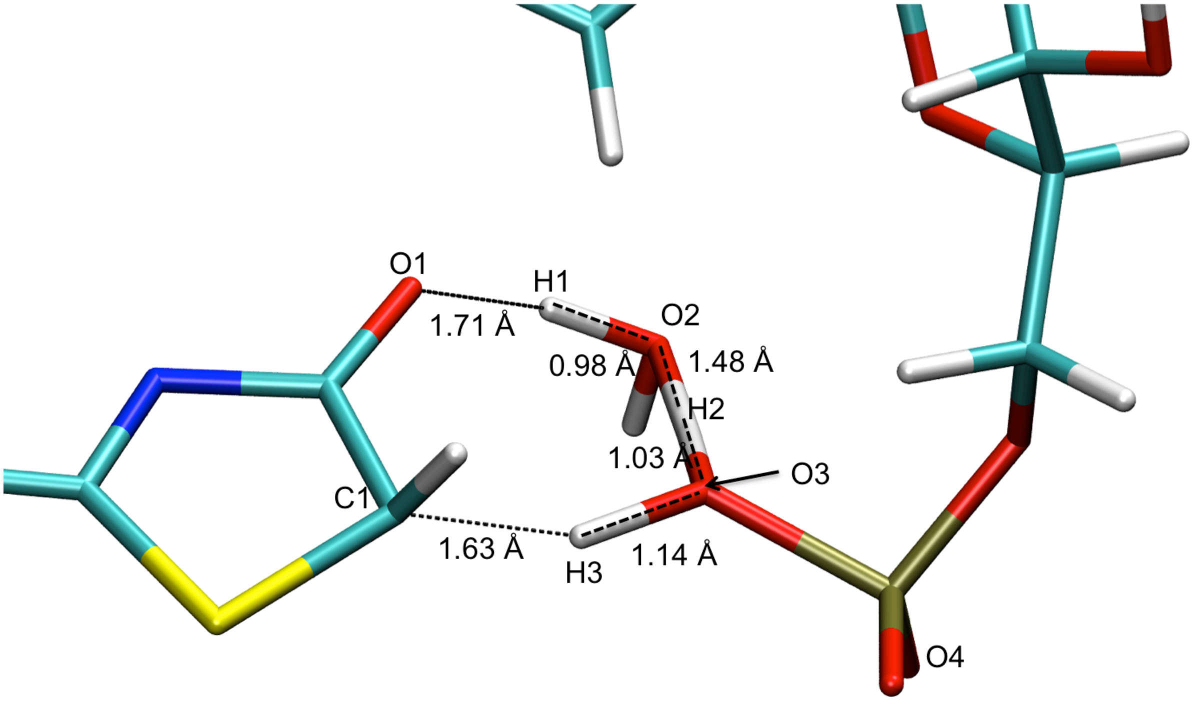

Supplementary Figure 3. Structure of TS_{GS}^{PCM}, displaying a 4 centers mechanism using PCM of water.

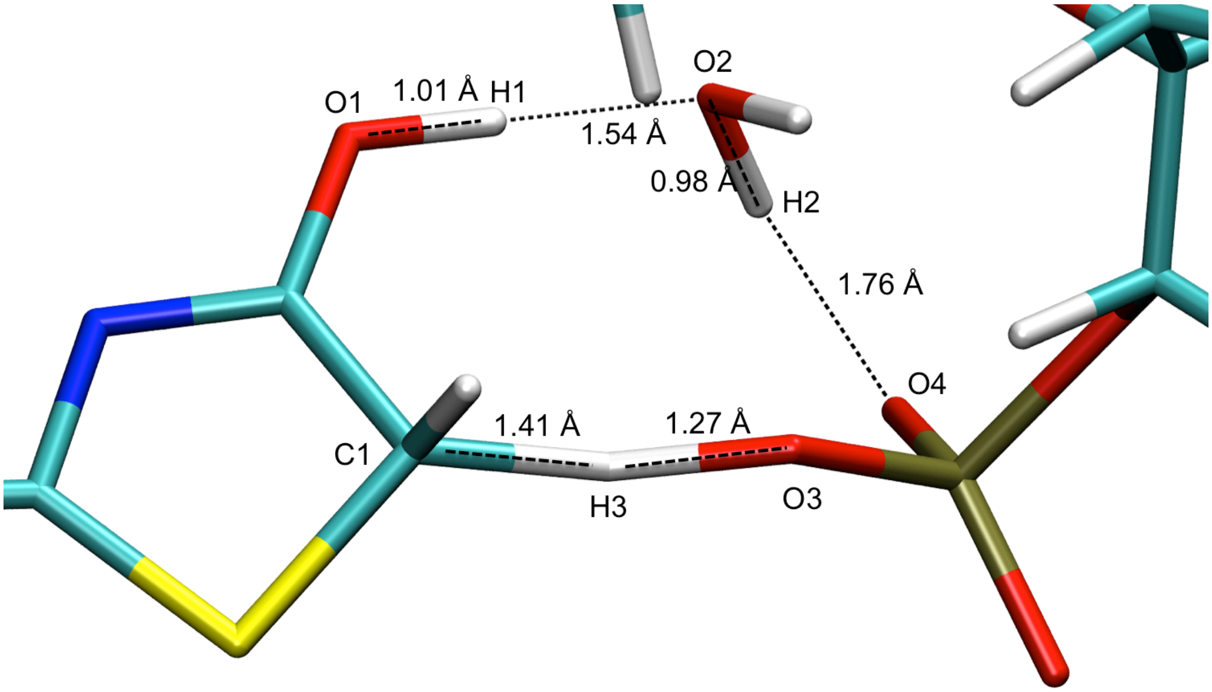

Supplementary Figure 4. Structure of TS_{S1}^{PCM}, displaying a 5 centers mechanism using PCM of water.

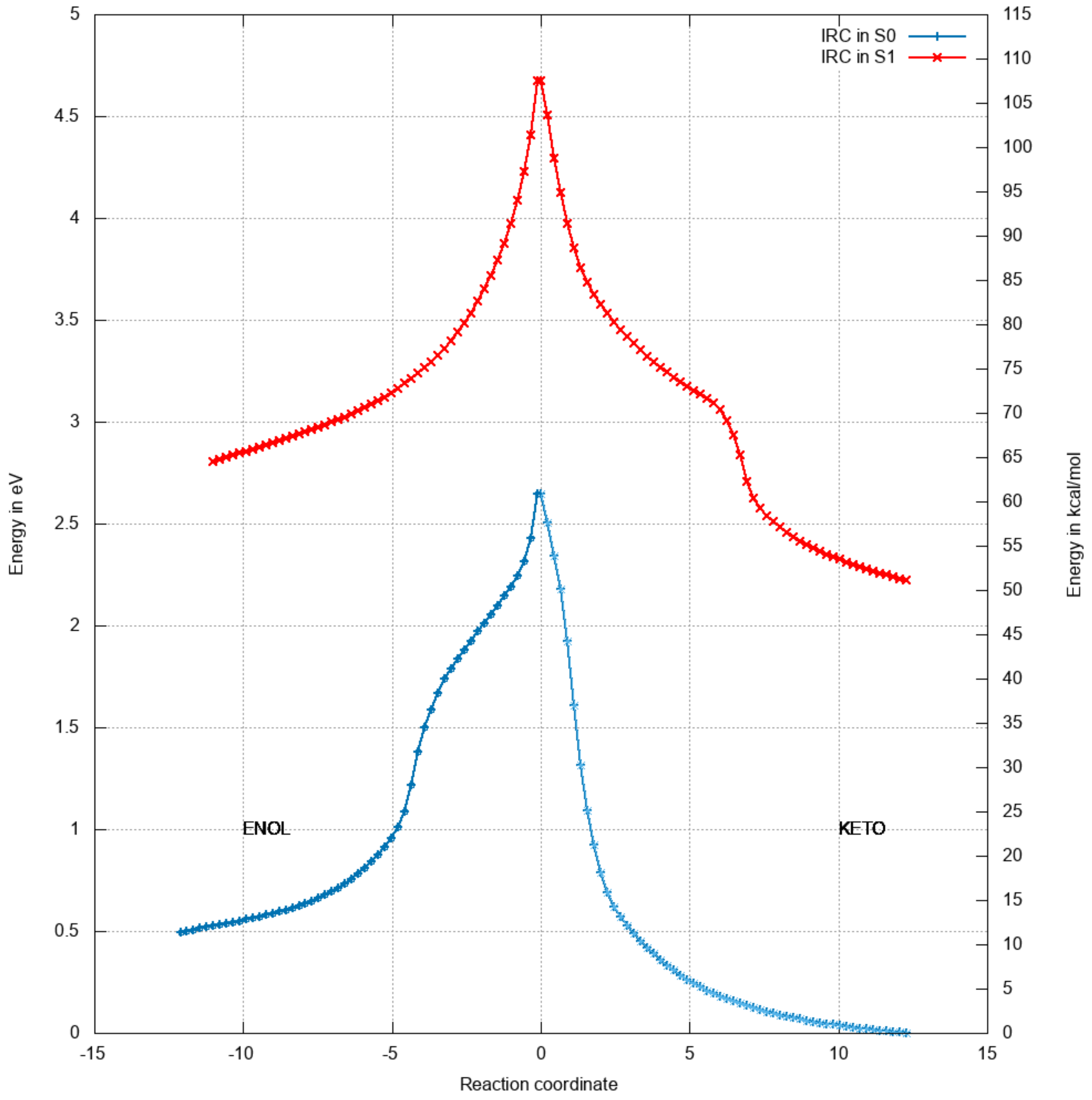

Supplementary Figure 5. Energetic profile of the keto-enol tautomerization starting from TS_{GS}^{PCM} and TS_{S1}^{PCM} at the reaction coordinate RC = 0. Superposition of GS in blue and S1 profiles in red. Positive RC values lead to the keto form while the negative ones to the enol form. The point at 0 eV corresponds to the lowest point of the energy profile that is to the keto form in the GS.

2 Note 1 on the obtaining of TS structure

To refine the TS structure obtained with the STQN algorithm, a Berny optimization has been performed with all the non-involved atoms in the tautomerization reaction blocked (See Supplementary Figure 1). In detail, it corresponds to the benzothiazole cycle of $OxyLH^{-}$, the ribose

and the adenosine part of AMPH. The number of imaginary frequencies was not tested along the TS optimization (using the *noeigentest* keyword in G09), which allows to locate TSs in large molecules. A frequency calculation was then performed to validate the found structure as TS, named TS_{GS}^{PCM}. Because of the freezing procedure, more than one imaginary frequency were found, with one of them being three orders of magnitude larger than the rest and corresponding to the keto-enol tautomerization.

3 Note 2 on the use of the algorithm local quadratic approximation (LQA)

The algorithm LQA (Page et al., 1990; Page and McIver, 1988) has been chosen because it is the only one available in G09/Tinker. In order to check the accuracy of the LQA algorithm, this algorithm together with the HPC (Hessian-based Predictor-Corrector integrator) (Hratchian and Schlegel, 2004) and the DVV (Damped Velocity Verlet Integrator) (Hratchian and Schlegel, 2002) algorithms were used to compute the IRC tried in implicit water calculations using PCM. Similar results have been obtained for the three algorithms (See Supplementary Figure 2), showing the suitability of the LQA to compute the IRC for QM/MM calculations. We have also used several keywords to compute the IRC in S1. As the second derivative of excited states are not included in G09, the keyword "gradient only" has therefore to be used. Again, no test of eigenvalues (IOP 1/11=1) was used. The geometric information of the guess structure was taken from the TS checkpoint file given as input (IOP 1/10=5), and the hessian matrix was computed at the beginning of the calculation (IOP 1/71=0).

4 Note 3 on the IRC using implicit solvent.

In the IRC computed in PCM, it is difficult to compare GS and S1, because the TS in GS corresponds to a 4 centers mechanism while it is a 5 centers one in S1. Moreover, the TS_{GS}^{PCM} is structurally closer to the keto form while for TS_{S1}^{PCM} it is close to the enol form. However, we can see that the keto form is more stable than the enol form in GS (about 12 kcal/mol) and S1 (about 13 kcal/mol). The computed barriers for the TS_{GS}^{PCM} are about 61 kcal/mol from keto to enol and 49 kcal/mol from enol to keto. For the TS_{S1}^{PCM} the barriers are about 56 kcal/mol from keto to enol and 43 kcal/mol from enol to keto.
For the TS_{S1}^{PCM}, we have not searched if a TS close to keto form can exist like inside the protein.

5 Note 4 on the computation of the $T_{e\_enol\_ref}^{prot}$ used in the main text.

The two electronic transition energies $T_{e\_enol\_ref}^{prot}$ and $T_{e\_keto\_ref}^{prot}$ are obtained according to this procedure. For $T_{e\_keto\_ref}^{prot}$ the calculations are done on the snapshot extracted from the MD1 and used as starting from the computation of the TS. For the $T_{e\_enol\_ref}^{prot}$ the snapshot used has been constructed like the one described in "Model setup" except the DLSA is decomposed into AMPH and enol-$OxyLH^-$. Thus, a MD simulation is conducted on this model and the lowest energy snapshot extracted and minimized at the MM level. The details used for the MD are the same as described in "MD simulations". For the two references, the emission energy (T_e) between the first singlet excited state (S1) and the ground state (GS), from geometries obtained in the S1 state, were computed using both the M06-2X and the B3LYP functionals using QM/MM calculations. The keto-$OxyLH^-$ or the enol-$OxyLH^-$ is defined as the QM part while the rest of the system (AMPH + water + protein) belong to the MM part. Finally, the basis set used is the 6-311G(2d,p). More details about the QM/MM calculations can be found in "QM/MM Setup".

6 References.

Hratchian, H. P., and Schlegel, H. B. (2002). Following reaction pathways using a damped classical trajectory algorithm. *J. Phys. Chem. A* 106, 165–169. doi:10.1021/jp012125b.

Hratchian, H. P., and Schlegel, H. B. (2004). Accurate reaction paths using a Hessian based predictor-corrector integrator. *J. Chem. Phys.* 120, 9918–9924. doi:10.1063/1.1724823.

Page, M., Doubleday, C., and McIver, J. W. (1990). Following steepest descent reaction paths. The use of higher energy derivatives with ab initio electronic structure methods. *J. Chem. Phys.* 93, 5634–5642. doi:10.1063/1.459634.

Page, M., and McIver, J. W. (1988). On evaluating the reaction path Hamiltonian. *J. Chem. Phys.* 88, 922–935. doi:10.1063/1.454172.
